# Supplementary material for: Identification of photoperiod-induced specific miRNAs in the adrenal glands of Sunite sheep (Ovis aries)
Source: Front Vet Sci. 2022 Jul 22;9:888207. doi: 10.3389/fvets.2022.888207 (PMC9354845; doi:10.3389/fvets.2022.888207)
Supplement: Supplementary file 1 [file Data_Sheet_1.ZIP › Supplementary file/miRDeep2 file.zip!/miRDeep2 file/Know_Structure/LP42A2/oar-mir-376d.pdf]

miRBase precursor : oar-mir-376d
Total read count : 182
oar-miR-376d read count : 175
remaining reads : 7

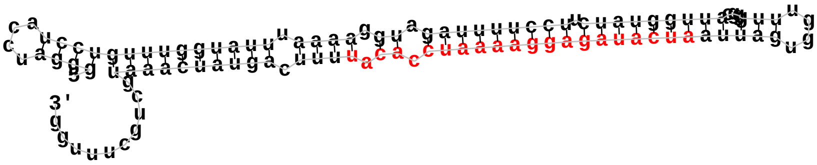

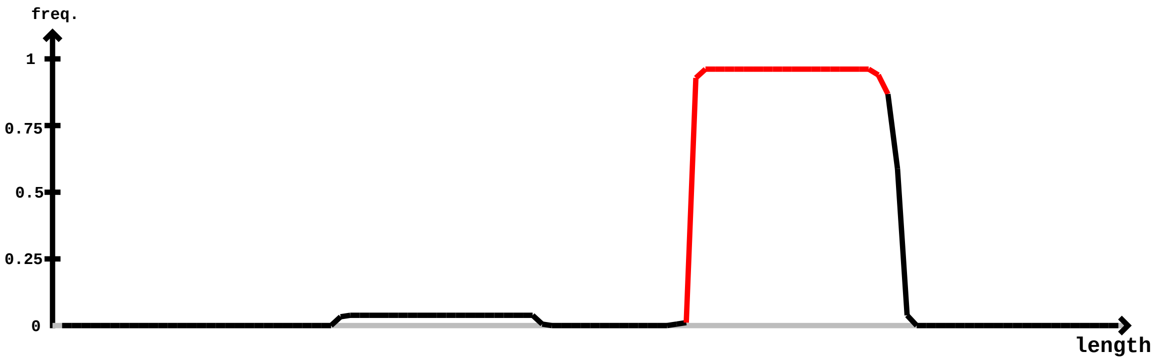

oar-miR-376d

```
5'- gggauccauccuguuugguauuuaaaagguagauuuuccuucuaugguuacggguuuggugauuaaucauagaggaaaauccacauuuucaguaucaaaugcugcuuugg  -3'   exp
    ((((....))))((((((((((.((((.((.(((((((((.(((((((((...(((....))))))))))))))))))))).)).)))).))))))))))..........   reads   mm   sample
    ............................guagauuuuccuucuaugguu.............................................................   5       0    seq
    ............................guagauuuuccuucuaugguua............................................................   1       0    seq
    .............................uagauuuuccuucuaugguu.............................................................   1       0    seq
    ...............................................................uaaucauagaggaaaauccac..........................   1       0    seq
    ................................................................aaucauagaggaaaauccaca.........................   1       0    seq
    .................................................................aucauagaggaaaauccac..........................   3       0    seq
    .................................................................aucauagaggaaaauccaca.........................   12      0    seq
    .................................................................aucauagaggaaaauccacau........................   51      0    seq
    .................................................................aucauagaggaaaauccacauu.......................   98      0    seq
    .................................................................aucauagaggaaaauccacauuu......................   3       0    seq
    ..................................................................ucauagaggaaaauccacauu.......................   2       0    seq
    ..................................................................ucauagaggaaaauccacauuu......................   4       0    seq
```
